# Assessment of Meat Content and Foreign Object Detection in Cattle Meatballs Using Ultrasonography, Radiography, and Electrical Impedance Tomography Imaging

**DOI:** 10.1155/2024/9526283

**Published:** 2024-08-01

**Authors:** Mokhamad Fakhrul Ulum, Maryani, Min Rahminiwati, Lina Choridah, Nurhuda Hendra Setyawan, Khusnul Ain, Utriweni Mukhaiyar, Fitra Aji Pamungkas, Jakaria, Agah Drajat Garnadi

**Affiliations:** ^1^ Division of Reproduction Obstetrics and Gynecology School of Veterinary Medicine and Biomedical Sciences Bogor Agricultural University, JlnAgatis, IPB Dramaga Campus, Bogor 16680, Indonesia; ^2^ School of Veterinary Medicine and Biomedical Sciences Bogor Agricultural University, JlnAgatis, IPB Dramaga Campus, Bogor 16680, Indonesia; ^3^ Division of Pharmacology and Toxicology School of Veterinary Medicine and Biomedical Sciences Bogor Agricultural University, JlnAgatis, IPB Dramaga Campus, Bogor 16680, Indonesia; ^4^ Faculty of Medicine Public Health and Nursing Universitas Gadjah Mada, Jl. Farmako, Sekip Utara, Depok, Sleman, Yogyakarta 55281, Indonesia; ^5^ Research Group of Biomedical Engineering Innovation Faculty of Science and Technology Airlangga University, Merr C Campus, Jl. Dr. Ir. H. Soekarno, Mulyorejo, Surabaya 60115, Indonesia; ^6^ Faculty of Statistics Bandung Institute of Technology, Ahmad Bakrie Building, 1st floor of Labtek VIII, Jln Ganesha No. 10, Bandung 40132, Indonesia; ^7^ Animal Husbandry Research Center National Research and Innovation Agency Cibinong Science Center, Jalan Raya Jakarta-Bogor, Cibinong, Bogor Regency 16915, Indonesia; ^8^ Departments of Animal Production and Technology Faculty of Animal Husbandry Bogor Agricultural University, JlnAgatis, IPB Dramaga Campus, Bogor 16680, Indonesia; ^9^ Faculty of Mathematics and Sciences Bogor Agricultural University, Jln Meranti, IPB Dramaga Campus, Bogor 16680, Indonesia

**Keywords:** cattle meatballs, electrical impedance tomography, food safety, foreign objects, physical hazards, radiography, ultrasonography

## Abstract

Meat content and physically hazardous contaminants in the internal section of meatballs cannot be detected by the naked eye or surface detectors. This study is aimed at analyzing the meat content of cattle meatballs and detecting foreign objects using ultrasonography (USG), digital radiography (DR), and electrical impedance tomography (EIT). Meatballs were produced using four different meat formulations (0%, 25%, 50%, and 75% meat) and three treatments (no preservative (control), borax, and formalin preservatives). Cast iron and plastic beads were used as models of foreign objects embedded in the samples. The echogenicity, opacity, and resistivity values of each sample were evaluated and compared across groups. The results showed that the shelf life of the control meatballs was shorter than that of meatballs with preservatives. The echogenicity and opacity values for the different meat formulations were hypoechoic in USG and grey in DR. USG was able to distinguish between control and preservative-treated meatballs but could not differentiate meat content and detect foreign objects. Conversely, DR effectively assessed meat content and detected iron-based foreign objects, while EIT showed higher resistivity values for iron and plastic beads compared to the meatball bodies.

## 1. Introduction

Several reports have shown that the consumption of processed animal products has increased along with economic growth. However, ensuring these products are safe, healthy, whole, and halal can be challenging. This condition highlights the need to develop methods to increase public confidence in food safety and to help assess food distribution according to the applicable requirements [1]. Meat is essential for maintaining body health, which can be fulfilled through livestock meat processing. The meat content of consumed food is crucial because inappropriately processed meat or substitution with nonmeat ingredients can harm consumers' health. Additionally, distributing food to distant areas often requires preservatives to maintain freshness and durability, which, if misused, can endanger consumers [2].

The determining of meat content in food products is still limited and typically involves identifying the meat source using various analytical methods [3]. For instance, protein levels in pork foods have been assessed using snapshot hyperspectral imaging sensor technology [4]. Physical, biological, and chemical hazards in food can endanger health and erode consumers' trust in food products [5]. Detection equipment is a critical component of the food production process, serving as the last defense against physical hazards [6]. Technologies such as X-ray [7], ultrasound [8], magnetic resonance [9], and microwave tomography [10] have been employed to detect hard materials like bone, plastic, glass, and metal. Additionally, near-infrared spectroscopy [11] and electrical impedance [12] can detect physical hazards in fluidic food. Electrical impedance tomography (EIT) is widely used in the industrial and biomedical fields due to its affordability, small size, simplicity, and lack of radiation, making it safe for use [13]. In industry, EIT is particularly useful for observing and describing materials during the mixing process in a vessel [14].

One of the most commonly consumed processed meat food products in Indonesia is meatballs [15], which are rich in animal proteins. Meatballs can be made from various meats, including beef, lamb, goat, chicken, fish, pork, and rabbit, and are highly susceptible to microbial spoilage. During manufacturing, biological contamination often occurs, leading to the use of dangerous preservatives like borax and formaldehyde (formalin), which pose significant health risks to consumers. Biological hazards or pathogens in food can be detected using nanoparticle sensors [16] or nanomaterial sensors [17]. Additionally, electronic noses have been reported to detect this adulterant practice [18]. Recently, both biological and chemical hazards were detected using paper-based microfluidics analytical devices [19].

There have been reports of incidents involving physical hazards in meat products in Brazil such as hamburgers (> 17%) and nuggets (> 9%) containing metal (9%), plastic (25%), and bone (27%) [20]. However, the incidence of physical hazards in meatballs is limited and challenging to determine. Internal physical hazards in the meatball cannot be detected by the naked eye or surface detectors [21]. Efforts to ensure safe food can include the nondestructive properties of ultrasound to analyze preserved food composition [22]. Other supporting tools for detecting specific tissues or materials include digital radiography (DR) [23], ultrasound (USG) [8, 24], and EIT [14, 25].

Therefore, this study is aimed at analyzing the meat content of meatballs and identifying foreign object models using USG, DR, and EIT. Additionally, the shelf lives of meatballs with and without preservatives were observed at room temperature (25°C –27°C) under controlled laboratory condition. The results of this study are expected to provide information to simplify the determination of meat content and foreign objects in products without damaging the meatballs.

## 2. Materials and Methods

### 2.1. Meatball-Making Procedure

The meatballs were divided into three groups based on preservative treatment: control (no preservative), borax, and formalin. Each group was further divided into four formulations with meat contents of 0%, 25%, 50%, and 75%. The primary ingredient was super ground beef B, containing 85% meat and 15% fat. The meatballs were prepared by weighing the ground beef according to its composition, followed by the addition of salt, pepper, seasonings, and tapioca flour. The total composition of flour and meat was 150 g. Preservatives were added in the form of 0.75 g borax for the borax group and 15 mL formalin for the formalin group. Sufficient water was added to achieve the desired consistency. The dough was then moulded into 250 mL plastic container, and foreign object models, such as iron and plastic beads, were inserted into the center of each meatball. The samples were steamed at approximately 70°C for 20 min and then cooled. The steaming process involved heating the samples in water at the temperature of 66°C–82°C.

### 2.2. Storage Observations

The storability of the meatballs was assessed based on five aspects: durability (weight loss), aroma, texture, colour, and the presence of microorganisms. Direct observations were performed daily until organoleptic changes and microorganism growth were detected, with a maximum observation period of 6 days. The weight loss was calculated using the following formula [26]:

(1) $$\begin{array}{c} \text{Weight loss}=\left[\frac{\text{initial weight}-\text{final weight}}{\text{initial weight}}\right]\times 100\% \end{array}$$

### 2.3. Nondestructive Imaging

#### 2.3.1. USG Imaging

Ultrasound image was conducted on the first day of storage using a Chison S6 ultrasound console (PT Mega Utama Medica, Palembang Indonesia) with a linear transducer set to a depth of 3 cm and a frequency of 9 MHz. Water was used instead of gel to facilitate scanning. The surface of the meatball was wetted with distilled water, and the transducer was attached to the wet surface. The transducer was moved along an imagined guide line crossing the embedded foreign object. Very high-frequency sound waves (ultrasound) passed through the body of the meatball. Some of the sound is reflected back to be captured by the transducer with a different level of sound wave reflection and then processed by the console into an image displayed on the monitor screen. The scanned sonogram is saved for further observation. The echogenicity of the scanned material appears as black (anechoic), grey (hypoechoic), and white (hyperechoic) in sonograms [27]. The captured sonogram images were saved for further analysis, and the echogenicity was evaluated using ImageJ (NIH, USA) software's greyscale histogram method [28].

#### 2.3.2. DR Imaging

Radiography was performed on the 4th day of storage. Meatballs were arranged based on their treatment, with the focal film distance (FFD) set to 100 cm, the milliampere second value was set at 6.20 mAs, and the peak kilovoltage was 54 kVp. Meatballs were placed on a plastic base on a table beneath an X-ray detector plate. The X-ray source is directed at the meatballs, and the resulting radiograph displayed on the monitor screen was evaluated for foreign bodies based on opacity using ImageJ software (NIH, USA). Radiographic interpretation was then performed by observing foreign bodies in the sample based on the opacity of their objects. Material opacity from the radiography appeared in black, white, and different degrees of grey. A black colour occurred when the X-rays burned the light film (in conventional radiography); however, in DR, these processes were changed by an electric film plate sensor that is sensitive to X-rays. Most degrees of high-level X-rays detected will be more black in images; further, partial or less X-rays detected by sensors will appear grey, and if there are no X-rays detected, the image will be white. Different material properties and their levels of X-ray absorption reveal different degrees of blackness of the film [27]. The greyscale histogram of ImageJ software (NIH, USA) was used to assess meat content in meatballs based on opacity [28].

#### 2.3.3. EIT Imaging

Resistivity data were collected on the 4^th^ to 5^th^ day of storage as the samples underwent organoleptic changes. Electrodes (electronic pins) were arranged in a straight line according to Wenner's configuration [29] with a spacing of 2 mm. The resistivity was determined using a set of four electrodes, namely, A, M, N, and B at Points 1, 2, 3, and 4, respectively, which were selected from a series of previously arranged 24 electrodes. Measurements started from the first electrode pin and continued until the end of the electrode series, completing the first level of imaging [30]. A 9 V DC power source was connected to a digital multimeter (Zotek, ZT98 CAT III, China), and the current (*I*) was then injected. Another digital multimeter was used for voltage (*V*) measurement. Subsequently, the voltage drop and current displayed on each multimeter were recorded. The electrode configuration was then returned to the starting positions of the survey line with the spacing increased to “2a,” and the second level of imaging was performed by moving the new arrangement until the end of the line. The spacing subsequently increased to 3a and 4a. It should be noted that the larger the electrode spacing, the deeper the imaging and the fewer the imaged points from that level [31]. Four investigation depth levels and 66 points inside the meatballs were imaged. The results obtained were calculated using Microsoft Excel software and converted into a .text file in Notepad software. The .text file was then entered into the Res2Dinv software to transform it into hot map imagery representing the resistivity value of the meatball component and foreign objects embedded within the meatballs [32].

### 2.4. Data Analysis

Qualitative analysis was performed on image data and described narratively. Quantitative data were expressed as mean ± standard deviation for each group. One-way analysis of variance (ANOVA) followed by Duncan's post hoc test was used to determine the differences between treatments at *P* < 0.05 using SPSS 26.

## 3. Results

### 3.1. Storage Observation

**Figure 1:**
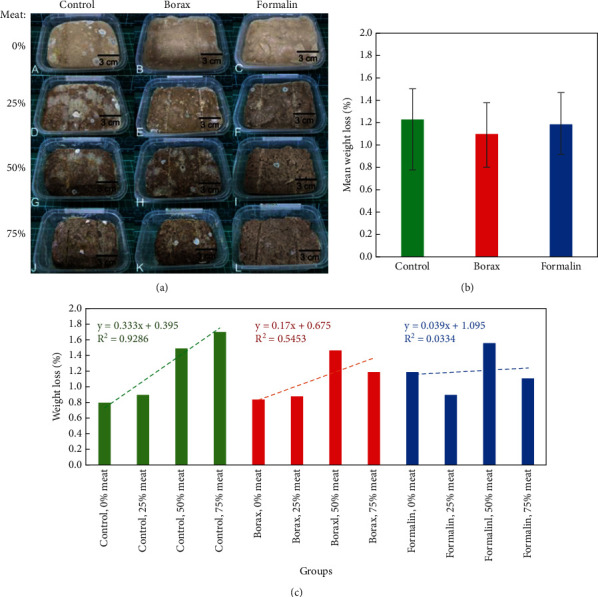
Representative image of (a) the observation of meatballs on the 5th day, (b) mean weight loss (%) of meatballs up to the 10th day, and (c) weight loss (%) of each meat content of meatballs in control, borax, and formalin groups. Contamination with microorganisms covered the surface of the meatballs during the storage period, making the discoloration indistinct. Shrinkage of shape and size occurred in the meatballs, representing weight loss (%), where the *R*^2^ of the control group (0.9286) was higher than that of the borax (0.5453) and formalin (0.0334) groups.

The aroma and texture observations indicated that control meatballs underwent faster changes, becoming septic more quickly than those treated with preservatives (Figure 1(a)). Normal meatballs had a characteristic aroma of meat and spices, whereas samples undergoing organoleptic changes developed a rotten smell. Microbial contamination was evident on the surfaces of control meatballs with 0% and 25% meat content, as well as borax (50% meat) and formalin (25, 50, and 75% meat) groups, causing indistinct discoloration.

Weight loss measurement over 10 days showed shrinkage in all meatballs. The mean weight loss was highest in the control (1.23 ± 0.45%) and formalin (1.19 ± 0.28%) groups compared to the borax group (1.10 ± 0.30%) (Figure 1(b)). The *R*^2^ for the control group (0.9286) was higher than that for the borax (0.5453) and formalin (0.0334) groups (Figure 1(c)). Notably, the highest shrinkage occurred in the control with 75% meat content, formalin (50% meat), and borax (50% meat) groups, with values of 1.71%, 1.56%, and 1.47%, respectively. The lowest shrinkage was observed in control (0%), borax (0%), and formalin (25% meat) samples, with values of 0.80%, 0.84%, and 0.90%, respectively.

### 3.2. Nondestructive Imaging

**Figure 2:**
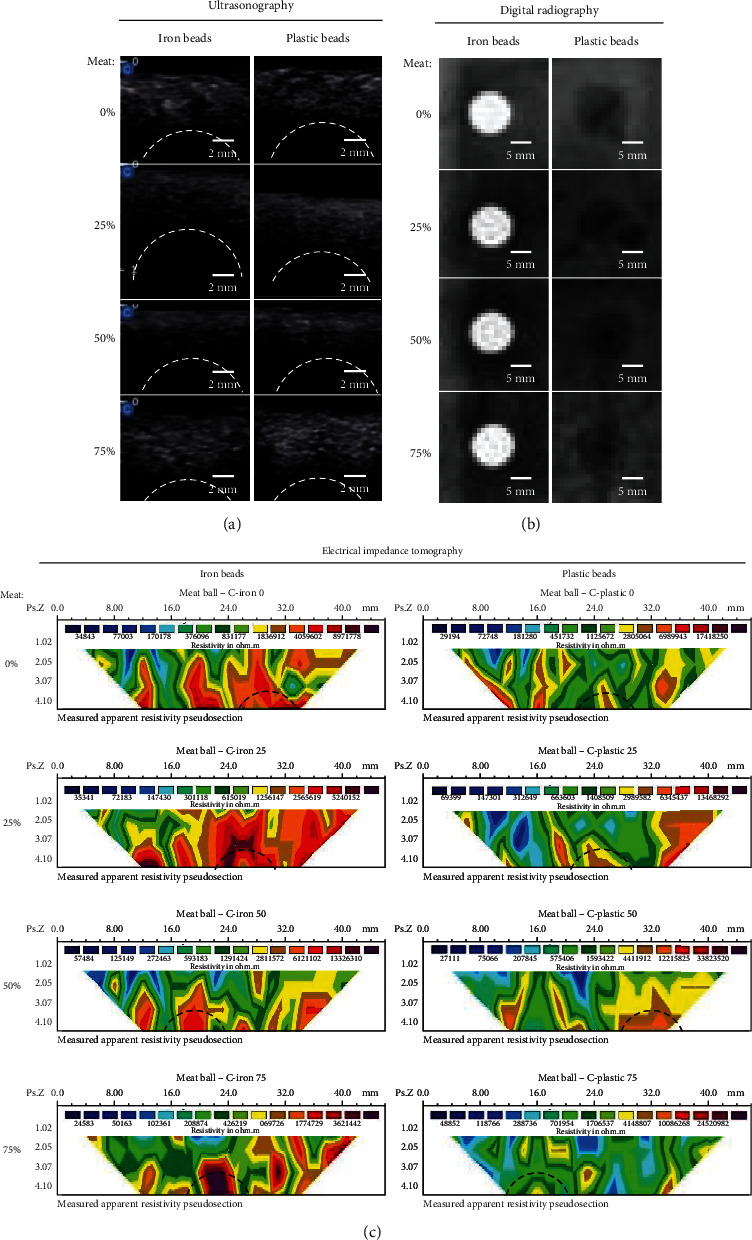
Representative image of iron and plastic beads on control meatballs using (a) ultrasonography, (b) digital radiography, and (c) electrical impedance tomography. (a) Foreign objects in the body of the meatballs did not appear on the ultrasonography images. (b) However, metal iron beads appear radiopaque in the form of a circular white colour, and plastic beads appear circular grey colour on digital radiography (a.u.). (c) Foreign objects appear as orange to violet colours with higher resistive values (*Ω*.m) than meatballs' bodies, which appear as dark blue to yellow colours as lower resistive value (*Ω*.m). *Note*: dotted white lines illustrate the position of foreign objects in the meatball.

Table 1 shows that borax and formalin samples with all meat contents (0%, 25%, 50%, and 75%) appeared more hypoechoic (dark grey) than the control group. The echogenicity of the meatballs in the ultrasound images was significantly different among the groups (*P* < 0.05). Despite these differences, no foreign objects were detected in the cross-sectional ultrasound images across all groups (Figure 2(a)).

DR revealed moderate opacity (grey) across all meatballs, with significant differences among the groups (*P* < 0.05) (Table 1). Meatballs with 0% meat content exhibited the highest opacity, while those with 50% meat content had the lowest. Foreign objects were clearly visible in the DR results: iron beads appeared radiopaque, and plastic beads appeared radiolucent (Figure 2(b)).

EIT imaging demonstrated that foreign objects (iron and plastic) exhibited higher resistivity values compared to the meatball bodies, which had lower resistivity values (Table 2 and Figure 2(c)). The resistivity contrast in the 2D cross-section indicated inhomogeneity in the meatball composition. Table 2 shows that the echogenicity of the foreign object was not analyzed because it did not appear on the sonogram. Furthermore, metal iron beads appear radiopaque in the form of a circular white colour, and plastic beads appear circular grey on DR (a.u.). Moreover, Wenner's configuration measurements of the trajectories of the control, borax, and formalin meatballs yielded varying resistivity values. The apparent resistivity pseudosection of the meatball samples had a region with a higher resistivity value (red to dark purple colours) compared to the surrounding area, which was suspected to be foreign objects, namely, iron and plastic. The resistivity contrast in the 2D cross-section shows uneven meatball body composition as shown in Figure 2(c).

## 4. Discussion

Detecting foreign objects in food through visual observation is challenging. This study successfully tested the detection of foreign objects in processed food products, specifically meatballs containing iron and plastic beads, using nondestructive techniques. Additionally, the effect of preservatives on the quality of ultrasound (USG), DR, and EIT images was examined.

### 4.1. Durability Tests

Durability tests demonstrated that meatballs experienced shrinkage over 10 days (Figure 1). The significant shrinkage was attributed to the diffusion of water content, weakening protein bonds in meat and amylose in tapioca flour during heating and storage at room temperature [33]. Control meatballs exhibited faster organoleptic changes, microorganism growth, and mucus appearance faster than those treated with preservatives. Borax-treated samples generally had a chewable texture lasting 3 days and an unnatural smell [34]. Formalin-treated meatballs have extended their shelf life, maintaining durable, nonsticky, nonwet, nonslimy, and nonmoldy texture with bright colours and unnatural aroma during 1–3 days of storage. Microorganism growth in food causes physical and chemical changes, such as decay [35].

### 4.2. Echogenicity and Opacity Analysis

The echogenicity values of meatballs varied based on their meat content and treatment (Table 1). Adding flour or water-soluble fibers increased echogenicity [36]. Previous studies reported muscles and fats as hypoechoic, whereas fluids were anechoic [37]. Control meatballs with meat 0%, 50%, and 75% meat content, along with 25% borax samples, had low echogenicity (anechoic) due to the higher water content during kneading. Factors such as water and fat accumulation in soft tissues influence echogenicity [27]. The use of borax and formalin resulted in a denser and chewable texture, impacting these parameters. Air trapped in the meatball body hindered the ultrasound's ability to capture deeper tissues, with black area on the sonogram indicating water presence (Figure 2(a))[38]. Consequently, foreign objects introduced into all samples were undetected on ultrasound images (Figure 2(a)).

DR revealed moderate opacity (grey or mixed) across all groups (Table 1). The opacity of primary ingredients like flour, meat, and water was similar, with water having an equivalent value to soft tissues (fat and muscle) due to their high-water composition [27]. DR could distinguish meat content based on opacity value but could not differentiate between meatballs with or without preservatives. Meatballs with 0% meat had the highest opacity, while those with 50% meat had the lowest due to equal meat and flour content, resulting in more radiolucent results. Higher opacity values in meatballs with 75% meat compared to 25% and 50% meat were attributed to the high-fat content of beef. Although fat is radiolucent, it can become radiopaque with increased thickness [27]. DR effectively observed foreign objects like iron and plastic beads in all samples, with metals appearing radiopaque and plastics more radiolucent [39].

### 4.3. EIT Analysis

High resistivity values were found in iron and plastic foreign objects in all samples except the control with 75% meat content (Table 2 and Figures 2(c)). Iron has a low resistivity of 1 × 10^−4^ *Ω*.mm [40], while plastic materials exhibit a resistivity of 1 × 10^21^ *Ω*.mm [41]. EIT images showed high resistivity in foreign objects due to factors like rust in iron beads, with electrical resistance and resistivity influenced by the degree of corrosion [40]. The cultivation process influenced by material and environmental factors (microbes, oxygen, water, electrolytes, and pH) also affects resistivity [42]. The 2D cross-section indicated uneven resistivity contrast, suggesting inhomogeneous meatball composition with air cavities causing high resistivity. Control samples with 75% meat did not show foreign plastics due to low-density distribution and position away from the projected current [43].

Overall, this study demonstrated that while USG was not effective in detecting foreign objects in meatballs, DR and EIT proved to be reliable methods for this purpose. The use of nondestructive imaging techniques offers a practical approach for assessing meat content and identifying foreign objects in food products, thereby enhancing food safety and quality control measures.

## 5. Conclusion

This study evaluated the effectiveness of ultrasonography (USG), DR, and EIT in determining meat content and detecting foreign objects in meatballs. The findings indicated that meatballs without preservatives had shorter shelf lives compared to those with preservatives. USG was not effective in detecting meat content or foreign objects in meatballs. However, DR successfully distinguished meatballs based on their opacity values and detected iron-based foreign objects. EIT provided images with inhomogeneous resistivity contrasts, allowing for the identification of foreign objects with distinct resistivity values compared to the meatball bodies.

These results suggest that DR and EIT are reliable nondestructive imaging techniques for assessing meat content and identifying foreign objects in meatballs. The use of these methods can enhance food safety and quality control measures by providing accurate and noninvasive means of detecting contaminants. Future research could focus on optimizing these imaging techniques for various food products and exploring the potential integration of multiple imaging modalities to improve detection accuracy and efficiency.

## 6. Limitation

While this study provides significant insights into the effectiveness of nondestructive imaging techniques (USG, DR, and EIT) in detecting meat content variations and foreign objects in meatballs, it is important to acknowledge certain limitations regarding microbiological assessments. The primary focus of this research was on developing and testing the imaging methods, rather than conducting comprehensive microbiological evaluations.

Visual fungal identification was employed as a preliminary indicator for the presence of contamination. However, we recognize the limitations of relying solely on this method. Comprehensive microbiological determinations, such as total viable count (TVC) and tests for pathogenic microorganisms (e.g., *Salmonella* and *E. coli*), are crucial for a thorough assessment of meat safety and quality [44, 45].

## Figures and Tables

**Table 1:** Echogenicity of meatballs on ultrasound and opacity of meatball body on digital radiography images.

**Group**	**Meat (%)**	**Ultrasonography**		**Digital radiography**		**Electrical impedance tomography**	
		**Echogenicity (pixel), (** **p** ** < = 0.001)**	**Category of echogenicity**	**Opacity (pixel) (** **p** ** = 0.003)**	**Category of opacity**	**Resistivity (** ***Ω*** **.Cm) (** **p** ** = 0.945)**	**Category of resistivity**
Control	0	56.50 ± 8.54^a^	Anechoic	131.66 ± 15.05^c^	Grey (mix)	67.66 ± 2.90^a^	Low
	25	59.73 ± 9.78^ab^	Anechoic	81.58 ± 9.84^ab^	Grey (mix)	79.17 ± 6.22^a^	Low
	50	56.73 ± 7.08^a^	Anechoic	77.88 ± 11.79^a^	Grey (mix)	67.73 ± 25.54^a^	Low
	75	57.69 ± 10.88^a^	Anechoic	90.58 ± 16.66^ab^	Grey (mix)	78.52 ± 56.90^a^	Low

Borax	0	76.75 ± 11.14^abc^	Hypoechoic	103.31 ± 11.53^ab^	Grey (mix)	75.69 ± 9.79^a^	Low
	25	70.92 ± 18.51^abc^	Hypoechoic	83.82 ± 11.96^ab^	Grey (mix)	97.22 ± 19.17^a^	Low
	50	81.23 ± 10.08^abc^	Hypoechoic	79.04 ± 15.98^ab^	Grey (mix)	98.34 ± 18.30^a^	Low
	75	85.73 ± 13.27^bcd^	Hypoechoic	106.46 ± 16.35^b^	Grey (mix)	88.66 ± 17.04^a^	Low

Formalin	0	108.00 ± 19.12^d^	Hypoechoic	102.21 ± 16.51^ab^	Grey (mix)	178.78 ± 28.78^a^	Low
	25	79.90 ± 3.35^abc^	Hypoechoic	83.37 ± 14.34^ab^	Grey (mix)	126.91 ± 35.44^a^	Low
	50	80.62 ± 18.55^cd^	Hypoechoic	76.28 ± 14.39^a^	Grey (mix)	182.73 ± 42.48^a^	Low
	75	66.61 ± 9.63^ab^	Hypoechoic	88.70 ± 17.95^ab^	Grey (mix)	136.77 ± 24.29^a^	Low

*Note*: Different superscript letters on the same line are significantly different (*P* < 0.05). The data was presented in an average form with a standard deviation (*x* ± SD). Category of echogenicity: anechoic/black (0–63); hypoechoic/dark grey (64–192); hyperechoic/bright (193–255). Category of opacity: radiolucent (0–63); grey (mix) (64–192); radiopaque (193–255).

**Table 2:** Echogenicity, opacity, and resistivity range values of foreign objects in meatballs on the image of ultrasonography, digital radiography, and electrical impedance tomography, respectively.

**Image**	**Foreign object**	**Control**	**Borax**	**Formalin**
Ultrasonography (echogenicity range value (a.u.))	Iron beads	n.a.	n.a.	n.a.
	Plastic beads	n.a.	n.a.	n.a.
Digital radiography (opacity range value (a.u.))	Iron beads	240.16–243.79 (radiopaque)	234.99–241.51 (radiopaque)	230.47–234.86 (radiopaque)
	Plastic beads	54.12–97.85 (radiolucent- grey)	64.98–84.25 (grey)	48.40–76.92 (radiolucent-grey)
Electrical impedance tomography (resistivity range value (*Ω*.mm))	Iron beads	4.97 × 10^7^ − 8.80 × 10^9^	1.50 × 10^8^ − 1.84 × 10^11^	1.84 × 10^8^ − 4.88 × 10^10^
	Plastic beads	5.50 × 10^7^ − 9.01 × 10^10^	8.23 × 10^7^ − 6.51 × 10^10^	8.33 × 10^7^ − 1.35 × 10^11^

*Note*: n.a.: not available data due to it cannot be detected on the US image; data is presented as the lowest to highest value (min–max); category of opacity: radiolucent (0–63); grey (mix) (64–192); radiopaque (193–255).

## Data Availability

All the data are presented in this paper. However, some of the data supporting our use in this paper were obtained from M's undergraduate thesis during his study at the School of Veterinary Medicine and Biomedical Sciences, Bogor Agricultural University (IPB University), under the supervision of M.F.U. and M.R.; M. is the author of this paper. Thesis data are deposited in the Bogor Agricultural University repository and are free-accessed online through https://repository.ipb.ac.id/handle/123456789/114365
